# Digital dashboards for oral anticoagulation management: a literature scoping review

**DOI:** 10.1007/s11239-023-02880-0

**Published:** 2023-08-18

**Authors:** Aaron S. Wilson, Darren M. Triller, Arthur Allen, Allison Burnett, Julie Ann Gouveia-Pisano, Allison Brenner, Barbara Pritchard, Charles Medico, Sara R. Vazquez, Dan M. Witt, Geoffrey D. Barnes

**Affiliations:** 1 University of Utah College of Pharmacy, Salt Lake City, UT 84112 USA; 2 Anticoagulation Forum, 17 Lincoln St, Suite 2B, Newton, MA 02461 USA; 3 VA Salt Lake City Health Care System, Salt Lake City, UT 84108 USA; 4 University of New Mexico Health Sciences Center, Albuquerque, NM 87102 USA; 5 Pfizer Inc, New York City, NY 10001-2192 USA; 6 Frankel Cardiovascular Center, University of Michigan, Ann Arbor, MI 48109 USA

**Keywords:** Anticoagulation, Dashboard, Population health, Stewardship

## Abstract

This scoping review summarizes the extent and characteristics of the published literature describing digital population management dashboards implemented to improve the quality of anticoagulant management. A standardized search protocol was executed to identify relevant manuscripts published between January 1, 2015 and May 31, 2022. The resulting records were systematically evaluated by multiple blinded reviewers and the findings from selected papers were evaluated and summarized. Twelve manuscripts were identified, originating from 5 organizations within the US and 2 from other countries. The majority (75%) described implementation in the outpatient setting. The identified papers described a variety of positive results of dashboard use, including a 24.5% reduction of questionable direct oral anticoagulant dosing in one organization, a 33.3% relative improvement in no-show appointments in an ambulatory care clinic, and a 75% improvement in intervention efficiency. One medical center achieved a 98.4% risk-appropriate venous thromboembolism risk prophylaxis prescribing rate and 40.6% reduction in anticoagulation-related adverse event rates. The manuscripts primarily described retrospective findings from single-center dashboard implementation experiences. Digital dashboards have been successfully implemented to support the anticoagulation of acute and ambulatory patients and available manuscripts suggest a positive impact on care-related processes and relevant patient outcomes. Prospective studies are needed to better characterize the implementation and impact of dashboards for anticoagulation management. Published reports suggest that digital dashboards may improve the quality, safety, and efficiency of anticoagulation management. Additional research is needed to validate these findings and to understand how best to implement these tools.

## Highlights

### Background

- Electronic health record-based dashboards have the potential to support high quality anticoagulation management for populations of patients
- A small number of health systems are known to have successfully implemented such dashboards
- The breadth of published evidence describing the development, implementation, and impact of anticoagulation management dashboards is unknown

### Findings

- Only a small number of centers have published manuscripts describing the use of digital dashboards
- Preliminary evidence suggests that digital dashboards positively impact care processes and clinical outcomes
- Prospective research is needed to characterize dashboard implementation and assess their impact on clinical outcomes

## Background and significance

Direct oral anticoagulants (DOACs) are the most commonly prescribed oral anticoagulants in the United States, due to their superior efficacy, enhanced safety profile, simpler dosing regimens, and the lack of frequent laboratory monitoring requirements as compared to warfarin [1]. Although approved indications vary by individual agent, multiple DOACs are considered first-line therapy for common thrombotic conditions (e.g., nonvalvular atrial fibrillation [NVAF] and venous thromboembolism [VTE]) [2, 3] and some are recognized as therapy options in clinical scenarios previously limited to injectable anticoagulants (e.g., postsurgical VTE prophylaxis, VTE prophylaxis in the medically ill) [4, 5]. Some are also approved for use in coronary and peripheral artery disease [6], further expanding the use of these agents.

However, anticoagulant use is associated with an increased risk of bleeding. This risk can be magnified in situations of inappropriate prescribing, drug-drug interactions, misuse by patients, and poorly managed care transitions [7]. Likewise, under-prescribing, prescribing inconsistent with FDA-approved labeling, and suboptimal patient adherence may contribute to avoidable thrombotic events, such as stroke [8, 9]. In fact, anticoagulants are the leading cause of adverse drug events in the emergency room [10].

In order to improve the quality, safety, and efficiency of the management of these high-risk agents, some health systems have developed, implemented, and evaluated digital “dashboards” that aggregate and analyze clinical data available for a population of patients prescribed the agents, identify potential clinical problems, and facilitate clinician intervention and tracking [11–15]. However, the breadth of use of such dashboards for the management of anticoagulants is uncertain. This literature review aims to evaluate and characterize the published literature describing the use of digital dashboards as clinical management tools for populations of patients indicated for anticoagulation treatment.

## Materials and methods

**Fig. 1:**
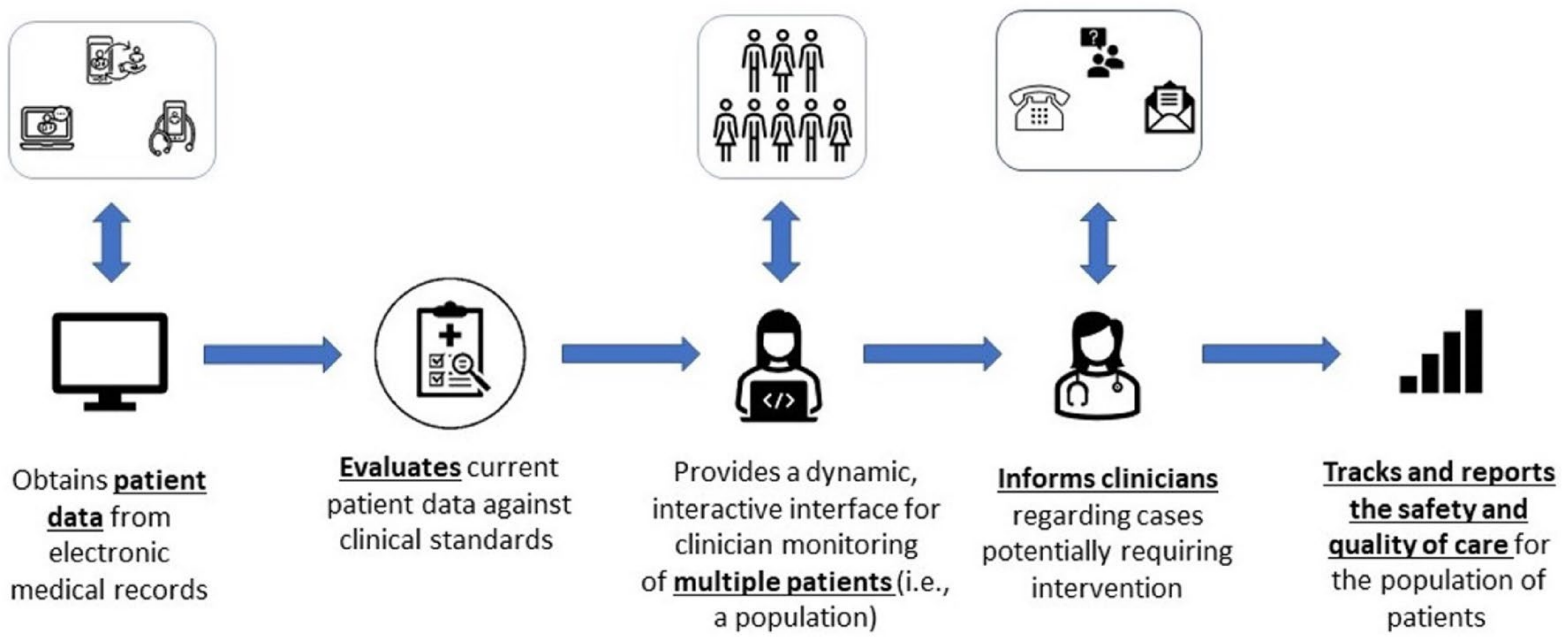
Dashboard definition. *Figure based on inclusion and exclusion criteria utilized by Tsang et al. [16]

**Fig. 2:**
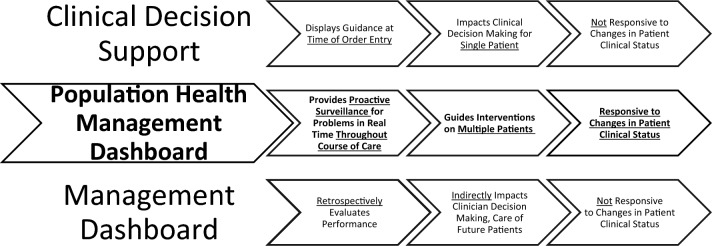
Comparing and contrasting digital tool characteristics

This scoping review sought to identify published manuscripts describing the development, implementation, and evaluation of digital dashboards for the management of anticoagulants. In the current context, “dashboard” refers to electronic health tools that aggregate digital clinical data, evaluate the data in the context of pre-identified standards, and present the data on multiple patients (i.e., populations) back to clinicians to support surveillance and guide clinical interventions for individual patients, when warranted (Fig. 1). This approach differs from clinical decision support, which generally influences care at the point of order entry, and quality or management reports, which are typically retrospective in nature (Fig. 2).

Among manuscripts known to the authors prior to performing the review, the terms “population health management tool”, “dashboard”, and others have been used, yet none had been used consistently or been established as the nomenclature standard. Therefore, an initial set of search terms was developed based on manuscripts known to the authors [11–15] and refined through iterative explorations of English language records in PubMed. Given the considerable heterogeneity in terminology and the existence of a prior systematic review that did not capture all papers known to the authors [16], a systematic, three-tiered search strategy was implemented (Appendix A). A search utilizing a narrow set of terms supplemental to the prior systematic review was performed for January 1, 2015 through January 31, 2021, and a search using an expanded term list was performed for manuscripts more recent than that systematic review (January 1, 2021–May 31, 2022). To account for heterogeneity in language, a manual search of tables of contents was also performed on 4 highly rated medical informatics and/or implementation journals for the complete time interval (January 1, 2015–May 31, 2022). Manuscripts that were not written in English, did not focus on the use of dashboards for anticoagulation management, or that described other types of digital tools to improve the quality or safety of anticoagulation management (e.g., electronic order sets, clinical decision support features) were excluded.

Identified records were imported into Rayyan systematic review software [17], after which duplicative and irrelevant records were removed based on evaluation of titles and abstracts by a single reviewer using pre-defined criteria (DMT or ASW). The remaining manuscripts then underwent blinded, 2-reviewer evaluations (DMT and ASW), with conflicts being resolved by a third reviewer (GDB). Retained manuscripts were then further evaluated by care setting (inpatient, outpatient), medications included (warfarin, direct oral anticoagulants (DOACs), or injectable anticoagulants), and whether the dashboard was utilized to intervene in individual patient care in real time. The protocol and search strategy were developed prior to initiating the literature search.

## Results

**Fig. 3:**
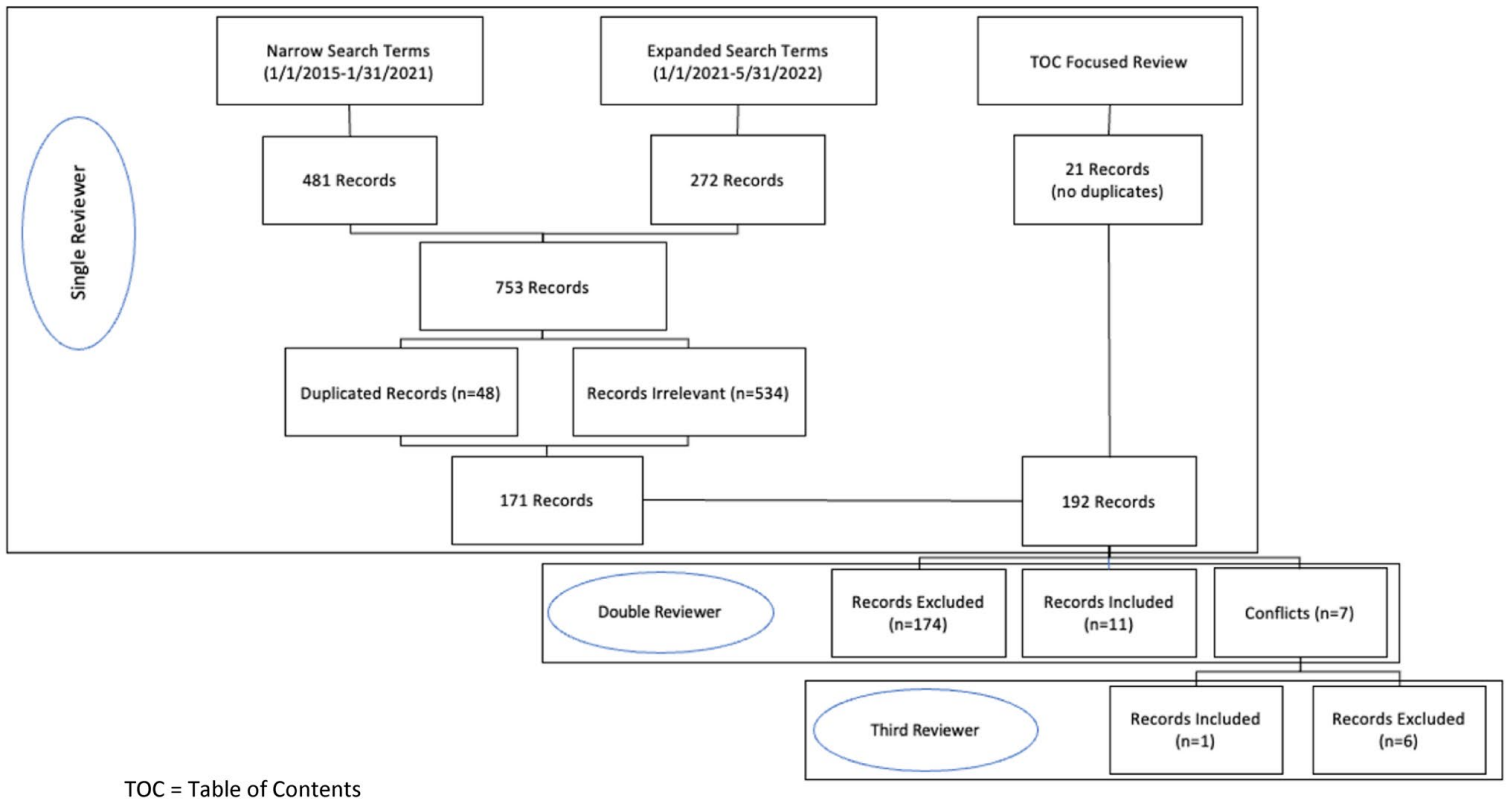
Flow diagram summarizing manuscript selection process

The initial detection phase for published manuscripts returned a total of 753 records (Fig. 3), which were then reduced to 192 records following title/abstract evaluation. The blinded, adjudicated review process resulted in a final total of 12 published manuscripts relating to the use of population health-focused dashboards for the management of patients indicated for anticoagulation therapy (Table 1) [11–15, 18–24]. The review noted several terms used to describe the tools used for anticoagulation management. These included “population health management tools”, “provider-level dashboards”, “integrated care applications”, “technology-enabled management platforms”, “electronic patient registries”, and “clinical surveillance tools”.

**Table 1:** Descriptions of included manuscripts

Author (Year)	Setting	Agent(s)	Real time patient intervention	Description
Michtalik et al. [24] (2015)	Acute Care^a^	All	No	Retrospective pre-post analysis of the impact of dashboard and a pay-for-performance program on VTE prophylaxis prescribing by hospitalists at an academic medical center. Monthly VTE prophylaxis compliance rates were 86% (95% CI: 85, 88), 90% (95% CI: 88, 93), and 94% (95% CI: 93, 96) during the baseline, dashboard, and combined dashboard/pay-for-performance periods, respectively. Compliance significantly improved with the use of the dashboard (p = 0.01) and addition of the pay-for-performance program (p = 0.01)
Lee et al. [23] (2017)	Ambulatory Care^b^	Warfarin	Yes	Retrospective pre-post evaluation of the impact of a dashboard on no-show rates among warfarin users at an urban, publicly funded clinic. For the cohort of 357 patients, the no-show rate decreased from 31 to 21%
Velencia et al. [15] (2019)	Ambulatory Care^c^	DOACs	Yes	Prospective evaluation of quality and character of interventions prompted by usual care (UC) and dashboard; UC generated 0.20 interventions per patient vs. 0.55 for dashboard (p < 0.001). Time to generate an intervention was shorter with dashboard (16 min) vs. UC (64 min)
Barnes et al. [12] (2020)	Ambulatory Care^d^	DOACs	Yes	Narrative manuscript describing the protocol for a study that will (1) evaluate the implementation of an EHR-based dashboard for medication management within the VHA, (2) guide the adoption in a set of four different health systems, and (3) evaluate the multi-center implementation effort
de Lusignan et al. [20] (2020)	Ambulatory Care^e^	DOAC, warfarin	No	Think aloud evaluation of a primary care atrial fibrillation management dashboard that supports (1) case ascertainment, (2) anticoagulation risk–benefit evaluation, (3) anticoagulant prescribing, and (4) correct dosing. AF management choices and prescribing choice sections were found to be the most useful indicators for clinical practice
Fontil et al. [21] (2020)	Ambulatory Care^b^	Warfarin	No	Narrative manuscript describing the protocol for a pre-post quasi-experimental design study to compare the impact of a dashboard management of warfarin on TTR compared with usual care at a large university-affiliated safety-net clinic
Rossier et al. [14] (2020)	Ambulatory Care^c^	DOACs	Yes	Retrospective, cross-sectional study of 40 VHA medical centers (20 Low, 20 High frequency dashboard users) with primary outcome of questionable DOAC dosing rate (QDR) between center types and secondarily across 8 subgroups; Results included higher QDR among Low use centers (17.5%, SD 4.2) than High use centers (13.2%, SD 5.2, p = 0.01) and across six of eight subgroups
Aboagye et al [18] (2021)	Acute Care^a^	All	No	Prospective cohort study of the impact of a personalized scorecard on VTE prescribing by medical trainees at an academic medical center. Mean percentage of risk-appropriate prophylaxis was significantly higher in both the wash-in (98.4%) and dashboard (98.4%) periods when compared to the prior scorecard method period (95.6%, p < 0.001)
Allen et al. [11] (2021)	Ambulatory Care^c^	DOACs	Yes	Narrative manuscript characterizing the development, implementation and spread of a DOAC management dashboard with the VHA system. Summarizes data from prior publications and posters on the initiative
Daniel et al. [19] (2021)	Acute Care^f^	DOAC, warfarin	Yes	Retrospective multicenter study of records of 56,761 patients receiving oral anticoagulants during a 6-month period before and after implementation of dashboard were reviewed for 31 hospitals within a nationwide health system. The oral anticoagulant ADE ratio decreased from 0.69% to 0.41% following dashboard implementation (p < 0.001)
Knaepen et al. [22] (2021)	Ambulatory Care^g^	DOACs	No	Validation and optimization of a multifaced app for patients with AF with a companion dashboard for clinicians. Included interviews of 15 healthcare professionals and 10 patients, and a pilot study of 1 month with 20 AF patients. Patient knowledge increased regarding the (1) disease state (71.9% to 87.5%, p = 0.013), (2) role of provider in periprocedural management (27.8% to 100%, p = 0.025), and (3) how to address missed doses (68.8% to 94.1%, p = 0.014). Use of clinician dashboard component was not described
Barnes et al. [13] (2022)	Ambulatory Care^d^	DOACs	Yes	Semi-structured interviews were conducted within the VHA (after dashboard implementation) and MAQI2 (prior to dashboard implementation). Among 45 interviewees, (32 in VHA, 13 MAQI2), 5 key determinants of implementation success were identified: (1) clinician authority and autonomy, (2) clinician self-identity and job satisfaction, (3) documentation and administrative needs, (4) staffing and work schedule, and (5) integration with existing information systems

*AF* atrial fibrillation, *ADE* adverse drug event, *EHR* electronic health record, *TTR* time in therapeutic range, *VTE* venous thromboembolism

^a^Johns Hopkins University, Baltimore, MD

^b^San Francisco General Hospital, CA

^c^US Veterans Health Administration (VHA)

^d^Michigan Anticoagulation Quality Improvement Initiative (MAQI2)

^e^Oxford Royal College of General Practitioners Research and Surveillance Centre, England, United Kingdom

^f^HCA Healthcare, Nashville, TN

^g^Jessa Hospital in Hasselt and Antwerp University Hospital, Belgium

The 12 retained manuscripts were published by 7 separate organizations, comprising both international (2/12, 16.7%) and US based institutions (10/12, 83.3%). The earliest detected manuscript was published in 2015, coinciding with our literature search cutoff date, though the majority were published from 2019 to present date (10/12, 83.3%). Most of the anticoagulation dashboard reports were used in the ambulatory care settings (9/12, 75.0%), with the remaining being from the acute care setting (3/12, 25.0%). These publications focused on management of DOACs, warfarin, and injectable anticoagulants, with some emphasizing specific anticoagulant agents and others not limiting their anticoagulant management tools to any specific agent. Injectable anticoagulants were discussed in 2 manuscripts (16.7%)—which focused solely on the use of anticoagulation management tools in the inpatient setting. Of the 12 manuscripts, only 7 (58.3%) described tools that were utilized for direct intervention in patient care.

These 7 studies described the significant, positive impact of dashboard use on a range of important care-related processes and clinical outcomes. Dashboard use was associated with a 24.5% relative reduction of questionable DOAC dosing among outpatients within the Veterans Health Administration (VHA) [14], a 9.3% relative improvement in guideline-compliant VTE prophylaxis prescribing in a tertiary care medical center [24], and a 33.3% relative improvement in no-show appointments in an ambulatory care clinic [23]. The use of a dashboard improved patient knowledge by 21.7% [22], improved the efficiency of clinician intervention by 75% [15], and improved clinician experience [20]. Dashboard implementation achieved a 98.4% risk-appropriate VTE risk prophylaxis prescribing rate among house staff at a tertiary care facility [18] and 40.6% relative reduction in anticoagulation-related adverse event rates [19]. One manuscript described the dashboard implementation strategy for an anticoagulation quality improvement initiative that spans the state of Michigan [12].

## Discussion

Published manuscripts describing the use of population health-focused anticoagulation management tools are limited but have been increasing in recent years. Overall, the available published literature suggests improved patient care with the use of anticoagulation-focused digital tools. These promising findings are welcomed and much needed, as anticoagulants continue to be among the agents most frequently associated with serious and preventable adverse drug events and the rate of such events is increasing [10]. As the population of patients eligible for anticoagulation continues to grow, impactful and efficient surveillance and clinical management methods are sorely needed.

However, while these early results are promising, the reports available to date are largely based on single center experiences and retrospective assessments whose designs limit their reproducibility and generalizability. For example, the reports include programs that included only medical residents [18], utilize an electronic health record limited in use to a single government agency [11, 13–15], focused on only a single agent [21], or characterized the results of a limited pilot [22]. Furthermore, none of the identified manuscripts describe improvement in clinical outcomes (e.g., bleeding, thromboembolic events). Rather, they focus on surrogate markers (e.g., appropriate medication prescribing) or process outcomes (e.g., clinician efficiency).

Prospective, multi-center studies reflecting recognized best practices in implementation science are needed to adequately control for confounding factors and to objectively assess important aspects of both the implementation process and the impact on well-defined process and outcome measures. Future studies of this type will not only add substantively to the evidence base but will also help consolidate the nomenclature used regarding such dashboards. They will also provide the detailed insights needed to guide administrative decision-making regarding investments in staff and necessary technology.

This literature review brings to light the research and practical use of dashboards that have been implemented within the field of anticoagulation management. Organizations such as the VHA and the Michigan Anticoagulation Quality Improvement Initiative (MAQI^2^) have demonstrated successful use of anticoagulation-focused dashboards [11–15]. The VHA and MAQI^2^ organizations have each provided in-depth insights into the impact of incorporating an anticoagulation management dashboard tool for managing anticoagulated patients in an outpatient setting. Each organization has equipped clinicians with tools designed to support evidence-based anticoagulation therapy and timely clinical interventions. The VHA data demonstrates that large scale implementation of anticoagulation dashboards is possible, given the successful integration of their anticoagulation-focused dashboard tool throughout the nation [25]. Furthermore, the MAQI^2^ data emphasize that implementation of dashboard resources has the potential to greatly improve drug management processes.

This literature review, while robust in design, was not without limitations. Despite increased interest in technology-based tools, there remains significant heterogeneity in nomenclature used for these tools within healthcare settings. This inconsistency in terminologies used among research centers may have limited the overall sensitivity of the PubMed queries. Additionally, this review was unable to incorporate information regarding tools that may already be in existence, but which have not yet been described in peer-reviewed medical literature. This limitation is further complicated by our inability to include manuscripts published in non-English languages, grey literature, and additional literature describing proprietary software.

## Conclusion

Early reports of initiatives utilizing digital dashboards to support safe and efficient anticoagulation management describe promising results that require validation in larger, prospective studies. Additional research is needed to understand how best to implement these tools within existing information technology systems and care models.

## Details of queries and search processes

### Scope of literature review

#### Description

Identify peer-reviewed published manuscripts describing the development, implementation, or clinical utility of digital tools that:

- Access and utilize clinical data available for a population of patients under the care of a responsible health care provider organization
- Identify and characterize a subset of patients prescribed or eligible to receive oral anticoagulants
- Support the centralized management of the subpopulation with the goal of increasing the quality and safety of AC-related care

#### Exclusion

- Manuscripts that describe electronic order sets, clinical decision support tools, ADE trigger tools and other EHR digital resources that are not accompanied by features to manage the subpopulation in aggregate
- Non-English

#### Time Interval: January 1, 2015–present

With the introduction of the Medicare Access and CHIP Reauthorization Act (MACRA) in 2015, the Medicare EHR Incentive Program, commonly referred to as meaningful use, was transitioned to become one of the four components of the new Merit-Based Incentive Payment System (MIPS), which itself is part of MACRA. https://www.healthit.gov/topic/meaningful-use-and-macra/meaningful-use

The 21^st^ Century Cures ACT was adopted in December of 2016, requiring developers of health IT, for their health IT to be certified, to meet certain requirements, including that the developer not engage in information blocking, which is preventing, discouraging, or interfering with the access, exchange, or use of information. https://www.congress.gov/bill/114th-congress/house-bill/34/

By including literature published since January 1, 2015, the review will encompass over 7 years of published evidence, including work affected by major federal incentive programs relating to health care quality and optimized use of electronic health records.

#### Database selection

After the evaluation of multiple commonly used reference resources, PubMed was selected as the sole database to be utilized for the literature review. PubMed covers the vast majority of medical and pharmacy journals, including those that published key manuscripts on dashboard use. It also encompasses 14 of the 15 highest-impact informatics journals. Other sources that were considered did not add substantively to PubMed’s holdings or failed to identify additional articles when search using initial keywords (Cochrane Library, Embase, International Pharmacy Abstracts, Web of Science).

Because the terminology utilized to describe clinical dashboards and the associated indexing process has not fully matured, the tables of contents of select health informatics journals will also be evaluated.

## Final approach to targeted literature review

Initial exploration and testing of the literature review process on the topic revealed several factors that have impact upon the final approach to be taken:

- A previous, high-quality systematic review has already been performed through January 2021 (Tsang et al.) and identified only one new manuscript on the topic
- Terminology and indexing processes in the subject are not mature, making automated searches less reliable

Considering these facts and recognizing that the present work is not intended to be a formal systematic review, a pragmatic 3-tiered approach to the final literature review will be taken:

1. For the time interval overlapping with that of the Tsang review (I.e., Jan 2015 thru Jan 2021, an automated search of PubMed will be performed using a narrow search term list [see Narrow Search Terms below]
2. For the time interval that does not overlap with the Tsang review (I.e., Jan 2021 to present) an automated search of PubMed will be performed utilizing an expanded search term list [see Expanded Search Term list]
3. To identify manuscripts in key health informatics journals that may not be fully indexed in PubMed, a direct search of their tables of contents will be performed. Journals include:
4. Implementation Science
5. Journal of the American Medical Informatics Association
6. Journal of Biomedical Informatics
7. Journal of Medical Internet Research

The records of all manuscripts identified through these processes will be aggregated in a single database for subsequent evaluation. A preliminary high-level review of their titles and abstracts will be used to identify papers that may potentially be of value to the search. A more thorough evaluation of these manuscripts will be performed by a pharmacist using the Manuscript Evaluation Tool as a guide.

An EndNote library will be created to organize the records of all identified manuscripts, with full text PDF files of each appended to each record. An annotated bibliography of the selected manuscripts will also be created. These resources will be utilized to inform subsequent phases of the project (e.g., survey development, expert forum).

### Narrow Search Terms (for Jan 2015–Jan 2021 search)

(2015/01/01:2021/02/01[Date—Publication] AND ("anticoagulant"[Title/Abstract] OR "anticoagulation"[Title/Abstract] OR "apixaban"[Title/Abstract] OR "atrial fibrillation"[Title/Abstract] OR "betrixaban"[Title/Abstract] OR "dabigatran"[Title/Abstract] OR "deep vein thrombosis"[Title/Abstract] OR "direct oral anticoagulant"[Title/Abstract] OR "DOAC"[Title/Abstract] OR "edoxaban"[Title/Abstract] OR "ischemic stroke"[Title/Abstract] OR "NOAC"[Title/Abstract] OR "novel oral anticoagulant"[Title/Abstract] OR "pulmonary embolism"[Title/Abstract] OR "rivaroxaban"[Title/Abstract] OR "target-specific oral anticoagulant"[Title/Abstract] OR "venous thromboembolism"[Title/Abstract] OR "warfarin"[Title/Abstract] OR "deep vein thrombosis"[Title/Abstract] OR "pulmonary embolism"[Title/Abstract] OR "ischemic stroke"[Title/Abstract] OR "venous thromboembolism"[Title/Abstract]) AND ("Population Health"[Title/Abstract] OR "dashboard"[Title/Abstract] OR "medical records systems, computerized"[MeSH Major Topic] OR ("electronic medical record"[Title/Abstract] OR "electronic health record"[Title/Abstract]) OR "population management"[Title/Abstract])).

### Expanded Search Terms (for January 2021–present)

2021/01/01: 2022/05/31[Date—Publication] AND ("anticoagulant"[Title/Abstract] OR "anticoagulation"[Title/Abstract] OR "apixaban"[Title/Abstract] OR "atrial fibrillation"[Title/Abstract] OR "betrixaban"[Title/Abstract] OR "dabigatran"[Title/Abstract] OR "deep vein thrombosis"[Title/Abstract] OR "direct oral anticoagulant"[Title/Abstract] OR "DOAC"[Title/Abstract] OR "edoxaban"[Title/Abstract] OR "ischemic stroke"[Title/Abstract] OR "NOAC"[Title/Abstract] OR "novel oral anticoagulant"[Title/Abstract] OR "pulmonary embolism"[Title/Abstract] OR "rivaroxaban"[Title/Abstract] OR "target-specific oral anticoagulant"[Title/Abstract] OR "venous thromboembolism"[Title/Abstract] OR "warfarin"[Title/Abstract] OR "deep vein thrombosis"[Title/Abstract] OR "pulmonary embolism"[Title/Abstract] OR "ischemic stroke"[Title/Abstract] OR "venous thromboembolism"[Title/Abstract]) AND.

("Population Health"[Title/Abstract] OR "dashboard"[Title/Abstract] OR "medical records systems, computerized"[MeSH Major Topic] OR "electronic medical record"[Title/Abstract] OR "electronic health record"[Title/Abstract]) OR "population management"[Title/Abstract] OR “anticoagulation stewardship”[tiab] OR “digital health”[tiab] OR “digital tool”[tiab] OR “data warehouse”[tiab] OR “implementation science”[tiab] OR “surveillance tool”[tiab]).

## Abbreviations

AF: Atrial fibrillation
DOAC: Direct oral anticoagulant
VTE: Venous thromboembolism
